# Nitrogen-Rich Polyaniline-Based Activated Carbon for Water Treatment: Adsorption Kinetics of Anionic Dye Methyl Orange

**DOI:** 10.3390/polym15040806

**Published:** 2023-02-06

**Authors:** Abdel-Basit Al-Odayni, Faisal S. Alsubaie, Waseem Sharaf Saeed

**Affiliations:** 1 Department of Restorative Dental Sciences, College of Dentistry, King Saud University, P.O. Box 60169, Riyadh 11545, Saudi Arabia; 2 Department of Chemistry, College of Science, King Saud University, P.O. Box 2455, Riyadh 11451, Saudi Arabia

**Keywords:** polyaniline, activated carbon, nitrogen-doped activated carbon, adsorption kinetics, methyl orange, water treatment

## Abstract

In the present work, a nitrogen-rich activated carbon (PAnAC) was prepared using polyaniline (PAn) as a precursor to represent one possible conversion of nitrogen-containing polymeric waste into a valuable adsorbent. PAnAC was fabricated under the chemical activation of KOH and a PAn precursor (in a 4:1 ratio) at 650 °C and was characterized using FTIR, SEM, BET, TGA, and CHN elemental composition. The structural characteristics support its applicability as an adsorbent material. The adsorption performance was assessed in terms of adsorption kinetics for contact time (0–180 min), methyl orange (MO) concentration (*C*_0_ = 50, 100, and 200 ppm), and adsorbent dosages (20, 40, and 80 mg per 250 mL batch). The kinetic results revealed a better fit to a pseudo-second-order, specifically nonlinear equation compared to pseudo-first-order and Elovich equations, which suggests multilayer coverage and a chemical sorption process. The adsorption capacity (*q_e_*) was optimal (405.6 mg/g) at MO *C*_0_ with PAnAC dosages of 200 ppm and 40 mg and increased as MO *C*_0_ increased but decreased as the adsorbent dosage increased. The adsorption mechanism assumes that chemisorption and the rate-controlling step are governed by mass transfer and intraparticle diffusion processes.

## 1. Introduction

Currently, water pollution has become a serious crisis and a great challenge for humanity. There are several natural and man-made activities that contribute directly or indirectly to water pollution. Water dirtiness may gradually increase as a result of humans’ activities, such as industrialization and civilization-related activities, which generate massive amounts of waste that is commonly discharged into water bodies and, subsequently, causes a lack of fresh water supplies [1]. About 300–400 megatons of industrial wastes are released into water bodies yearly and uncontrollably introduce various pollutants to aquatic environments, most of which are toxic to living creatures [2]. Such wastes may combine solid matter, harmful gases, toxic chemicals, and microbiological organisms. However, regarding their sources and compositions, pollutants affect our health and environment differently [3]. In particular, chemicals are known to be major water pollutants. They are present in various types, including dyes, heavy metals, pesticides, chemical compounds, cosmetics, pharmaceutics, and hormones.

Dyes, however, are among the common chemical substances largely discharged into water bodies and are considered the most dangerous pollutants due to their potential carcinogenic and mutagenic effects. Furthermore, their solubility in water affects the environment aggressively by changing the water chemistry, reducing the amounts of dissolved oxygen by obstructing sunlight penetration into the water, driving unwanted effects on aquatic creatures, and drastically disturbing their biosystems [3].

Industrially, azo dyes, including methyl orange (MO) and mordant black 17, are the most popular category and are used in diverse applications, such as in the textile, paper, and printing industries. Further, they possess the highest production rate among all types, representing about 60% of all dyes. The threat of these dyes arises, as aforementioned, from their solubility in water. In addition, as their molecular weights increase, their distribution in water also increases. Hence, their degradation rate decreases. MO is a well-known colorant, being one of the most used industrial azo-based dyes. Additionally, it is a popular laboratory pH indicator, working as a weak acid with a red-to-yellow color-change range at pH 3.1–4.4 [4].

To eliminate dyes from water, massive studies were carried out to find an efficient solution. Thus, different technologies such as adsorption, filtration, precipitation, ion exchanges, flocculation, coagulation, and ozonation were applied. Adsorption is a highly remarkable method that is used in dye removal due to its simplicity, low costs, and excellent efficiency [5,6,7,8]. Materials such as clays, zeolites, activated carbon, carbon nanomaterials, polymers, biomass, and agricultural wastes were commonly investigated as adsorbent candidates. However, efficient adsorbents must have suitable properties to be applied in the field of dye removal. Thus, materials with high surface areas, low toxicity, renewability, low waste formation, low costs, and high stability are typically favorable [7,9,10,11].

Since the eighteenth century, carbon-based materials have been used to adsorb unwanted constituents from gases and solutions [12]. Activated carbon (AC) is one carbon-based material that has been comprehensively studied and applied in adsorption. It is well known for its high porosity, enormous capacity, and low costs. Such features encourage its use for water treatment. Nevertheless, AC is a reachable material due to its simple preparation methods and multivarious abundant precursors, including wood, biomass, bones, industrial byproducts, and any other carbon-rich materials [12,13]. Basically, AC preparation can be approached by the activation (chemically or physically) of precursors (e.g., biomass). Chemical activation may proceed via the impregnation of the source with oxidizing/hydrating reagents. Then, it is carbonized at between 400 and 900 °C. On the other hand, the physical activation process consists of two stages performed in two different furnaces, starting with carbonization under the ambient atmosphere, followed by activation under an oxidizing atmosphere at between 800 and 1100 °C [13].

This work aimed to synthesize a new, efficient, nitrogen-rich activated carbon (denoted PAnAC) adsorbent based on PAn as a precursor and to investigate its kinetic adsorption process for the removal of MO as an anionic azo-dye model. The PAnAC adsorbent was fully characterized and applied in a batch kinetic adsorption process at various doses and MO concentrations. The performance was assessed using various kinetic models and is discussed in reference to the physicochemical and structural properties of the adsorbents.

## 2. Materials and Methods

### 2.1. Materials

Aniline (C_6_H_7_N) (Ani, 99%), ammonium persulfate ((NH_4_)_2_S_2_O_8_) (APS, 98%), and potassium hydroxide (KOH) (pellets, 85%) were procured from Alfa Aesar, Karlsruhe, Germany. Hydrochloric acid (HCl) (~36%) and absolute ethanol (EtOH, 99.5%) were purchased from Fisher Chemical, Loughborough, U.K. Methyl orange sodium salt (C_14_H_14_N_3_NaO_3_S) (MO, 99.8%) powder was obtained from BDH Chemicals Ltd., Poole, England, UK. All reagents were used as received unless stated otherwise. Distilled water was used throughout the experimental process.

### 2.2. Preparation of Adsorbents

The adsorbent, PAnAC, was prepared using a previously described method [6]. Typically, a total amount equal to 0.08 mol of aniline monomer was charged into a precooled (in an ice bath of about 5 °C) 1L container of a 0.1 M HNO_3_ aqueous solution and stirred for 30 min. To the monomer solution, 30 mL of a cold aqueous APS (0.12 mol) solution was added dropwise as an oxidative agent (initiator). The polymeric product immediately appeared; however, the reaction was left overnight, for completeness, at room temperature (23 ± 2 °C). The precipitate was filtered and sequentially washed with ethanol and water to obtain PAn. PAnAC was prepared using KOH as a chemical activating agent. Therefore, PAn was thoroughly mixed with KOH at a mass ratio of 1:4, respectively. Typically, KOH was individually powdered using a pestle and mortar and immediately mixed with the corresponding PAn quantity, with additional homogenization using a plastic spatula. Subsequently, the mixture was subjected to carbonization in a horizontal Carbolite MTF 12/38/250 tube furnace (Walf Laboratories, Hope, England, UK) under the following conditions: nitrogen atmosphere, heating ramp rate of 3 °C/min, activation time of 2 h, and activation temperature of 650 °C. The obtained carbon PAnAC was washed several times using 0.5 M HCl and water until neutrality, followed by drying in an oven at 100 °C overnight.

The surface charges of the adsorbents under investigation were determined using a pH drift method described elsewhere [14]. Initially, a pH series between 1 and 11 was prepared using a buffered solution of 0.1 M NaNO_3_; the targeted pHs were obtained by the addition of 0.1 M of either HCl or NaOH and denoted as initial pH (pH_i_). To each 15 mL of these solutions, 15 mg of the adsorbent was added and shaken intermittently for 24 h at room temperature. Adsorbent-free samples were also prepared and treated similarly as references. Subsequently, the adsorbents were filtered and analyzed to determine their final pHs (pH_f_). The pH measurements were conducted using an Orion 3 Star benchtop pH meter from Thermo Scientific (Beverly, MA, USA). Finally, the pH at which the surface net charge of the adsorbents was zero (pH_PZC_) was determined by plotting pH_i_ vs. pH_f_.

### 2.3. Adsorbate

A stock solution of MO at a concentration of 500 ppm was prepared in distilled water, from which the working solutions were obtained using a dilution method. The pH values of the solutions were determined using a digital pH meter as described above. The concentration of the dye was measured using a U-2910 double-beam ultraviolet/visible (UV/Vis) spectrophotometer (Hitachi, Tokyo, Japan) at room temperature. The wavelength at which the dye adsorption was highest (λ_max_) was determined first by scanning the full spectrum between 200 and 800 nm against distilled water as a blank, and it was found to be 465 nm. All colorimetric measurements were performed at this λ_max_. Then, the concentrations were calculated based on the Beer–Lambert law, with reference to a standard curve obtained using 5, 10, 15, 20, and 25 ppm (*R*^2^ = 0.9815).

### 2.4. Characterization

The structural and morphological properties of the adsorbent were evaluated using various techniques. The Brunauer–Emmett–Teller (BET) specific surface area was obtained based on the nitrogen physisorption capacity at 77 K using a Gemini VII2390 V1.03 apparatus (Micromeritics, Norcross, GA, USA), with the instrument operating in single-point and multipoint modes. Prior to analysis, the samples were degassed for 3 h at 150 °C. The electron micrographs were acquired using a scanning electron microscope (SEM) (JSM-6360 LV, JEOL, Tokyo, Japan). A thermogravimetric analysis (TGA) was performed using a Mettler Toledo TGA/DSC 1 Star system (Columbus, OH, USA). The sample was heated from 25 to 800 °C at 25 °C/min under a nitrogen flow of 20 mL/min. The Fourier transform infrared (FTIR) spectra of the adsorbents before and after adsorption were recorded using a Nicolet iS10 (Thermo scientific, Madison, WI, USA) with an attenuated total reflection (ATR) diamond crystal accessory over the range of 4000–500 cm^−1^, with a resolution of 4 cm^−1^ and a total of 16 scans per spectrum.

### 2.5. Modeling of Adsorption Kinetics Processes

Pseudo-first-order (PFO), pseudo-second-order (PSO), Elovich, and intraparticle diffusion (IPD) are the most applied kinetic models for the optimization of sorption process. Thus, in this study, linear and nonlinear forms for MO adsorption onto the targeted activated carbons were adopted to fit the experimental data. The kinetics profiles of the adsorbent PAnAC were compared, with variations in the initial adsorbate MO concentration (*C*_0_ = 50, 100, and 200 ppm) and adsorbent dose (20, 40, and 80 mg) at a fixed stirring rate (150 rpm), solution volume (250 mL), and temperature (23 ± 2 °C). The mathematical expressions of the applied models are given in Table 1. The accuracy of the fit of an adsorption model to the experiential data is typically assessed based on the magnitude of correlation coefficient (*R*^2^) values. Thus, when approaching unity, the predicted values become closer to the experimental data, which can help in the assessment of the process mechanism and the applicability of the system.

#### 2.5.1. PFO Model

The Lagergren kinetic equation (PFO) is the most widely used for describing a kinetic system. The model is based on the assumption that one site is available for each adsorbate molecule. Moreover, the solute uptake with time is directly proportional to the difference between the equilibrium concentration of the solute and its adsorbed amount with time [15,16]. The linear and nonlinear equations are given in Table 1. The parameters were calculated from the corresponding plot with respect to time, and thus the rate constant *k*_1_ (min^−1^) was determined.

#### 2.5.2. PSO Model

The PSO linear and nonlinear kinetic models are given in Table 1. The models assume two sites on the adsorbent for each adsorbate molecule [17,18] and suggest the dominance of the chemisorption process [19]. Moreover, the reaction rate is dependent on the amount of adsorbate on the surface of the adsorbent [20]. The parameters can be obtained by plotting the experimental *q_t_* or *t*/*q_t_* against *t*. The adsorption rate constant *k*_2_, as well as the initial rate (*h*, mg/(g·min), can be obtained by solving the PSO equations, as shown in Table 1.

#### 2.5.3. Elovich Model

The Elovich equations for linear and nonlinear adsorption kinetics are also given in Table 1. The model assumes that the adsorbent sites are energetically heterogenous and that neither desorption nor interactions between the adsorbed species could substantially affect the kinetics process at low surface coverage [21]. However, the energy of adsorption increases linearly with the surface coverage according to Arrhenius equation [22]. The parameters 𝛼 and 𝛽 are related to the initial rate and the extent of surface coverage, respectively, as given by the model. The latter parameter can be used to assess the chemisorption process.

#### 2.5.4. IPD Model

The adsorbate transfer through the internal porous structure and its actual diffusion in the adsorbate can be followed using the IPD model given by Webber and Morris, as seen in Table 1. The model constants *k_id_* and *C* describe the rate constant and boundary layer, which are useful for identifying the reaction pathway and adsorption mechanism and predicting the rate-controlling step. In short, if the linear slope passes through the origin, the adsorption is entirely governed by the IPD method. Otherwise, the plot may show multilinear regions, which suppose that the adsorption process is controlled by a multistep mechanism [23].

## 3. Results and Discussion

### 3.1. Adsorbent Characterization

#### 3.1.1. Surface Morphology and Charge

**Figure 1:**
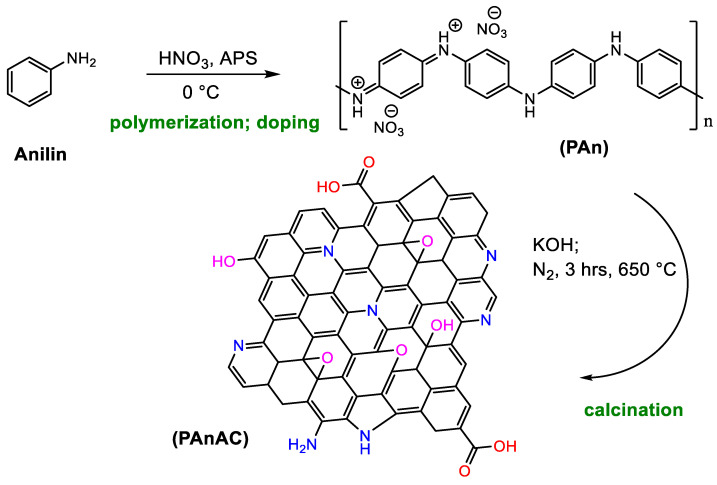
Schematic presentation of PAn and PAnAC synthesis and proposed structures.

Figure 1 is a schematic presentation of PAn and its corresponding nitrogen-doped graphene oxide (PAnAC) structures. According to the elemental analysis results (Table 2), PAnAC has high amounts of the heteroatoms nitrogen and oxygen, about 18 and 20 wt%, respectively. This suggests the presence of high number of active sites in the adsorbent and thus a high expected adsorption capacity.

**Figure 2:**
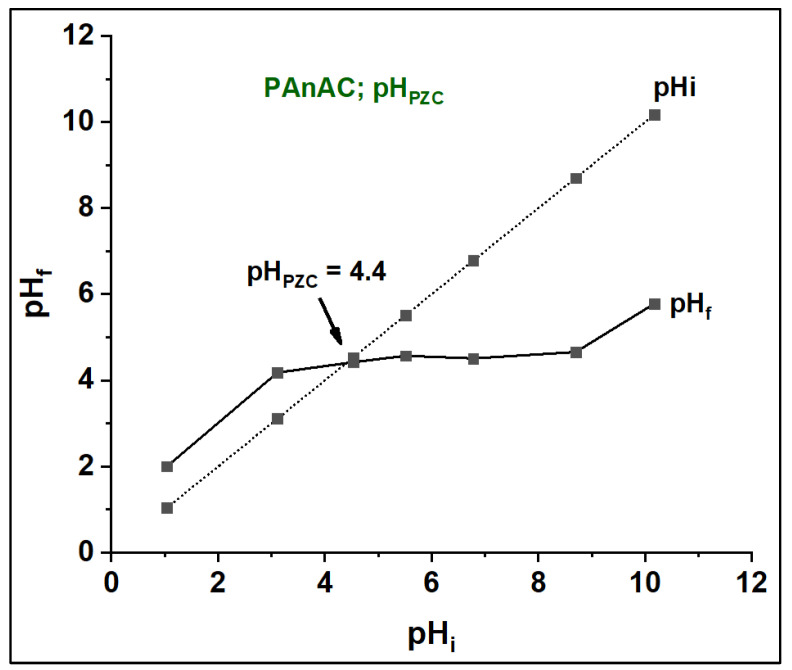
Point of zero charge (pH_PZC_) for PAnAC adsorbents.

The predicted surface charges or, more strictly, the pH at which the estimated surface charge is zero (pH_PZC_) is shown in Figure 2. This property was analyzed using the basic pH drift method, and accordingly the pH_PZC_ of PAnAC was found to be 4.4. Typically, the surface is neutral at pH_PZC_, below which the surface charge is positive and above which the surface charge is negative. Basically, MO is a crystalline salt that dissociates in water into an anionic MO moiety and cationic counterparts (commonly Na^+^). Thus, at a pH below pH_PZC_, the adsorbent positive surface supported attraction with MO as a negative species, and its efficiency seemingly decreased as the pH value developed up to pH_PZC_, possibly due to reduced electrostatic repulsion at low pH values. As the adsorption experiments were carried out without pH adjustment (pHs were measured before the addition of the adsorbents at 20 ± 2 °C for MO concentrations of 50, 100, and 200 ppm and were found to be 6.69, 6.25, and 6.13, respectively) and the pH_PZC_ of PAnAC is quite low, its adsorption capacity was high.

**Figure 3:**
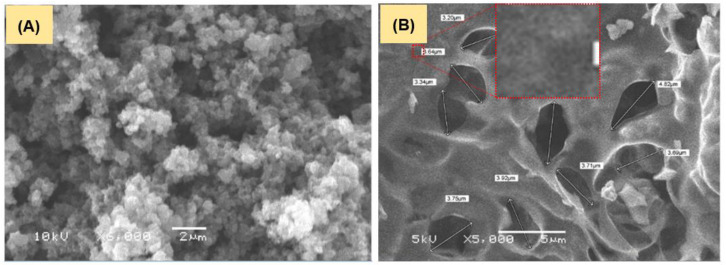
SEM micrographs of (**A**) PAn and (**B**) PAnAC.

Figure 3 shows SEM images of the as-synthesized PAn and PAnAC. The PAn image reveals the typical nanosphere morphology of PAn, which further fused together into microsphere structures, a morphology similar to the previously reported morphology [27]. The SEM micrograph of PAnAC presents a rough structure with a peel with a surface with quite large irregular-shaped pores with a mean internal diameter of 3.7 μm. Despite the low resolution of the adsorbent image in Figure 3B, the zooming shown in the inset could predict a nanocarbon structure and pores.

#### 3.1.2. FTIR Analysis

**Figure 4:**
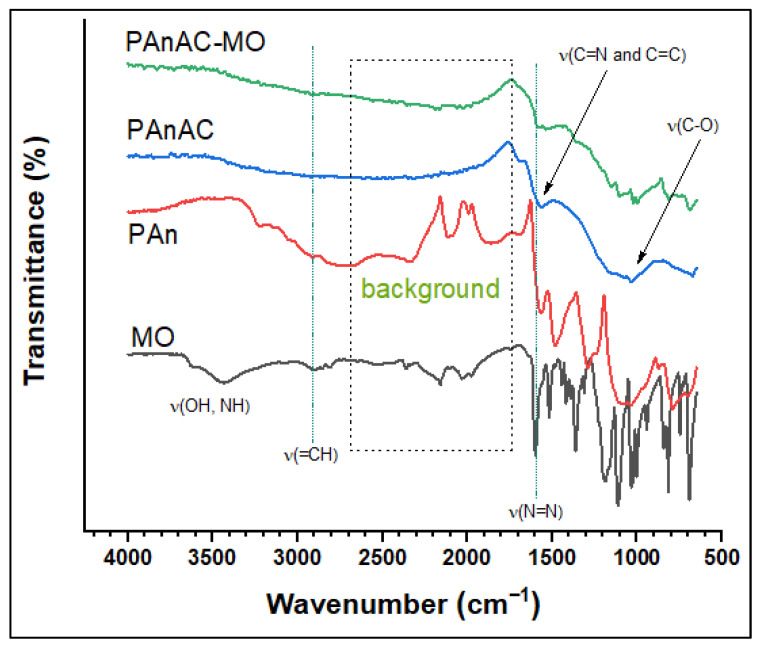
FTIR spectra for methyl orange (MO), polyaniline (PAn), activated carbon (PAnAC), and PAnAC-loaded MO (PAnAC-MO).

The spectra of PAn, PAn-based activated carbons before (PAnAC) and after the adsorption of MO (PAnAC-MO), and MO are shown in Figure 4. The spectra of MO, PAn, PPyAC, and PPyAC–MO agreed with the literature [4,28,29]. The MO spectrum displayed peaks at 3626 and 3431 cm^−1^ for adsorbed water–OH, at 3030 cm^−1^ (=CH) and 2900–2811 cm^−1^ (CH_3_) for stretching vibrations, and at 1519 cm^−1^ for C–H bending. The aromatic structure of MO was confirmable via ring deformation modes at 1034, 1005, and 846 cm^−1^, while benzene substitution was assured by the characteristic peak at 816 cm^−1^ [28]. The Azo moiety was proven by the presence of stretching bands at 1599 (-N=N-) and 1112 cm^−1^ (-C-N). The sulfonic nature was affirmed by peaks at 1363 and 692 cm^−1^, assigned to S=O and -C-S- stretching vibrations, respectively. In the spectrum of PAn, the peaks at 1538 and 1456 cm^−1^ were typical of C=N stretching vibrations.

The characteristic peaks of PAn could be seen at (cm^−1^) 3219 (NH stretching, reported for protonated form [30]), 3048 (=CH stretching), 2917 (CH_3_ stretching), 2328 (C=NH-C, immonium), 1569 and 1484 (C=N and C=C stretching in quinoid and benzenoid rings, respectively), 1289 and 1241 cm^−1^ (C-N-C and C-N stretching), 1036 and 876 cm^−1^ (C-H bending; its broadening may indicate contributions of peaks from the dopant NO^3−^), and 792 cm^−1^ (1,4-disubstituted phenyl ring [31,32]). After adsorption, the absorption of C=N (1557 cm^−1^) and C-O (1035 cm^−1^) were shifted to 1539 and 1157 cm^−1^, respectively, suggesting contributions of nitrogen and oxygen groups in the adsorption mechanism. Furthermore, the additional peaks on the fingerprint region were assigned to MO and supported its attachment to the adsorbent [4].

#### 3.1.3. Thermal Analysis

**Figure 5:**
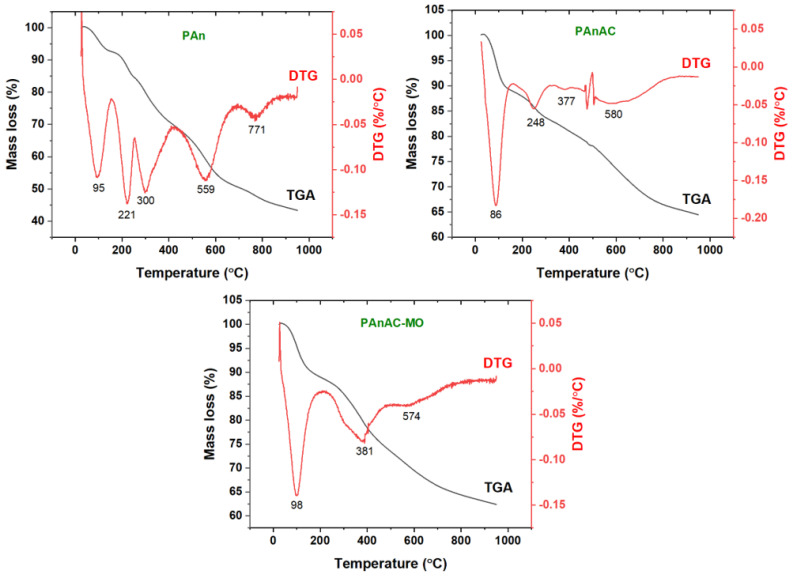
TGA and DTG curves of PAn, PAnAC, and PAnAC-MO.

A thermal analysis of PAn, PAnAC, and PAnAC-MO was also carried out. The corresponding thermograms and the predicted decomposition steps are given in Figure 5 and Table 3. As can be seen, the decomposition curves can be generalized in five steps for polymers, four steps for PAnAC, and two steps for MO-loaded PAnAC. The initial TGA decomposition step, with mass losses of 7.4–11.2, was predictably due to the evaporation of volatiles, including adsorbed water and gas molecules, which peaked at 95, 86, and 98 °C in the DTG curves of PAn, PAnAC, and PAnAC-MO, respectively. The next step could be assigned to weak functional groups in the target materials, which were slightly absent in the PAnAC and MO-loaded PAnAC, showing DTG peaks at 221, 248, and 381 °C, respectively, and were supported by the extra steps for PAn. The analysis also revealed residual masses of 43.4, 64.5, and 62.3 %, respectively, at 950 °C, which is consistent with the chemical structures. After adsorption, the trend in the residual carbon content remained the same. However, the values were higher after adsorption, which may have been due to the higher content of adsorbed volatiles in the PAnACs, as assessed by the mass loss percentage in the first step. Additionally, the observed high residue of PAnAC before adsorption may have indicated an incomplete carbonaceous property, suggesting high amounts of heteroatoms.

### 3.2. Adsorption Studies

#### 3.2.1. Effect of pH

**Figure 6:**
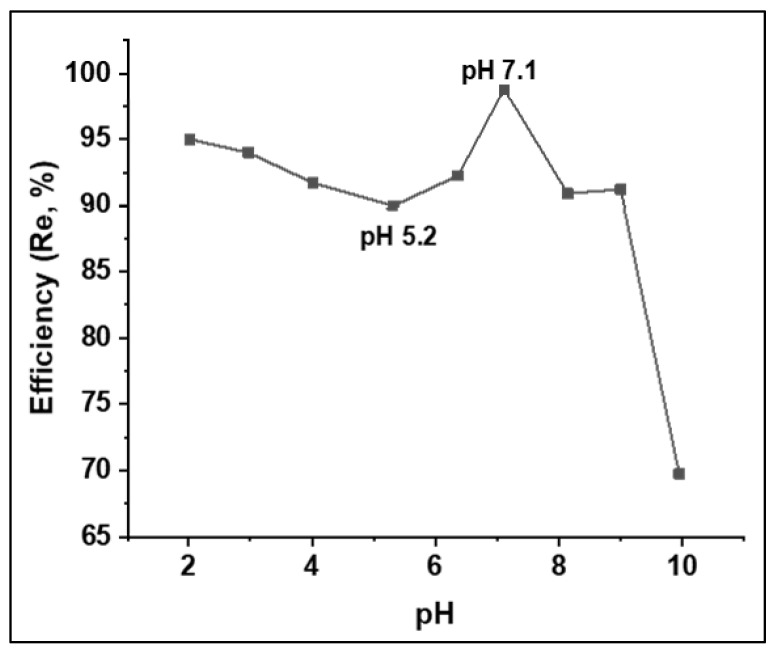
Solution pH effect on adsorption efficiency of PAnAC adsorbents for MO uptake.

The effect of the solution pH was analyzed under the following conditions: adsorbate MO initial concentration: 200 ppm, MO volume: 25 mL, adsorbent dose: 15 mg, shaking speed: 150 rpm, temperature: 25 °C, and contact time: 24 h. The pH solution was varied between 2 and 10. The results are the averages of two independent experiments. The dependency of the sorption process on the solution pH is depicted in Figure 6. It was found that the adsorption had minimum and maximum values at pHs around 5 and 7, respectively. However, pH 7.1 was close to the untreated pollutant solution (pH 6.4). Therefore, the next experiments were performed without pH adjustments. As can be seen in Figure 6, the adsorption efficiency steadily decreased with a pH increase from 2 to 5.2, then increased and peaked at about pH 7.1 before decreasing rapidly over the remaining pH range of 7.1–10. At a low pH, the adsorbent was highly protonated. Thus, electrostatic attraction with the anionic dye was expected, which decreased as the pH developed up to pH_PZC_. However, with continued increases in pH, the efficiency increased, and this was mostly due to the contributions of other mechanisms, including physical interactions, rather than electrostatic interactions. This case was again observed beyond the peak of pH 7.1; that is, a drop in efficiency occurred due to the electrostatic repulsion between the negatively charge surface and the anionic dye. Furthermore, it is likely that the competition between dye molecules and hydroxide ions for adsorption onto PAnAC may explain the sharp decline in the adsorption in the basic range of pHs [33].

#### 3.2.2. Kinetic Studies: Effect of Adsorbate Concentration

The adsorption kinetic courses of MO at initial concentrations of 50, 100, and 200 ppm onto PAnAC at a dosage of 40 mg were evaluated over the predetermined contact time of 0–180 min at a fixed agitation speed of 150 rpm, a temperature of 23 °C, and a pH of 6.4. In all experiments, 250 mL batches of MO were used, and a 3 mL portion was withdrawn at predefined intervals, then centrifuged, measured for the remaining MO concentration, suspended again, and returned to the main adsorption solution. The results were modeled using the linear and nonlinear equations of PFO, PSO, and Elovich kinetics for comparison. In addition, IPD was utilized to help with the adsorption mechanism assessment.

**Figure 7:**
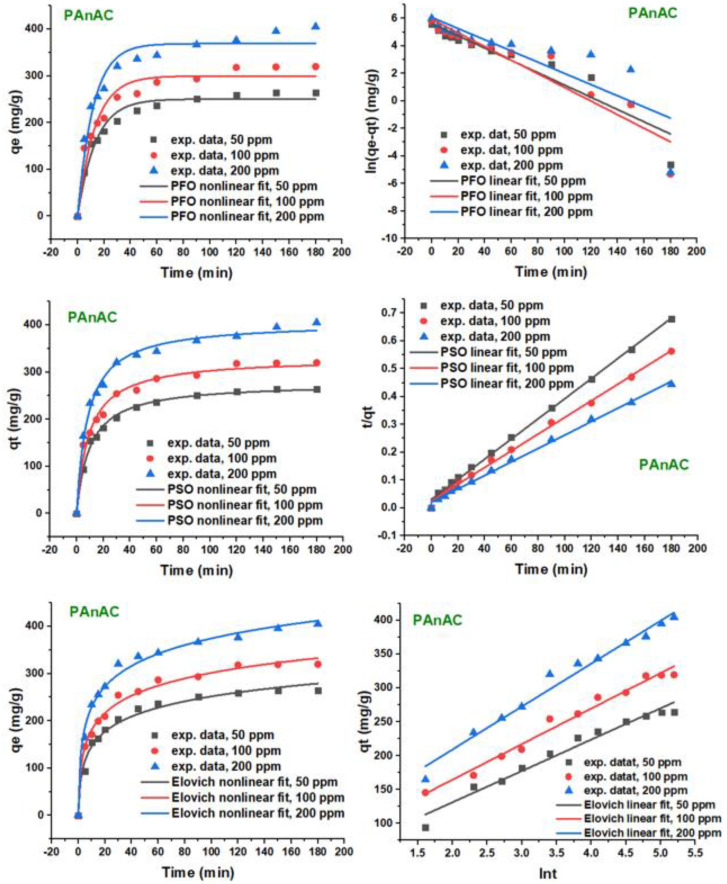
Linear and nonlinear plots of PFO, PSO, and Elovich. Conditions: MO adsorbate concentrations: 50, 100, and 200 ppm; MO volume: 250 mL; adsorbent PAnAC dose: 40 mg; pH of solutions: 6.4; agitation speed: 150 rpm; temperature: 23 °C; contact time: 0–180 min.

**Figure 8:**
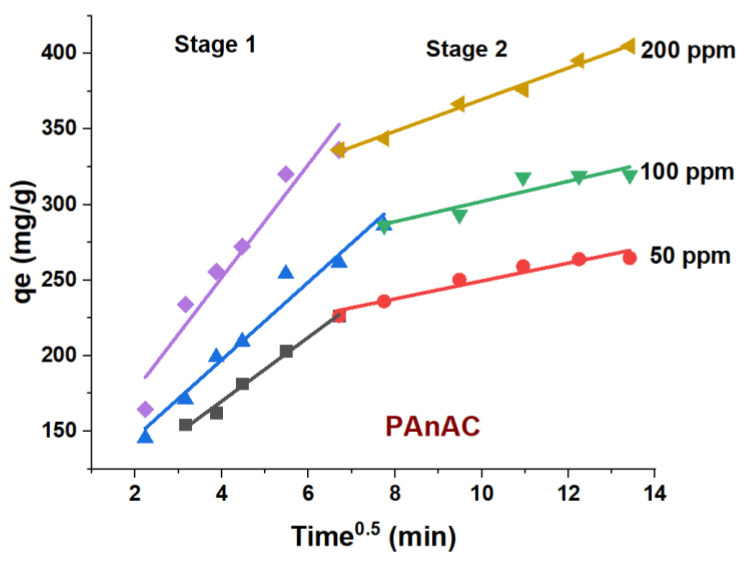
Intraparticle diffusion plot for adsorption of methyl orange (MO) onto PAnAC adsorbent at MO concentrations of 50, 100, and 200 ppm. Conditions: as given in Figure 7.

Figure 7 and Figure 8 show the applied kinetic models at varied MO initial concentrations, and the corresponding parameters are summarized in Table 4 and Table 5. As can be seen, the adsorption rates in the initial period, assessed before 30 min, for the three *C*_0_ values were fast, where more than two thirds of the adsorbents were occupied with MO molecules. This reveals that the adsorption occurred on the adsorbent surface and was governed by charge interactions [4]. Following the first stage, the adsorption developed slowly but quickly reached the equilibrium at ca. 60 min.

By increasing the MO adsorbate concentration from 50 to 200, the uptake rate become slower, and the equilibrium point was delayed accordingly. The capacity was found to be increased with an initial MO concentration increase. For example, the experimental capacity (*q_e_*_,exp_) of PAnAC was increased from 264.7 to 405.0 (mg/g) as MO *C*_0_ increased from 50 to 200 ppm. These results are in accordance with the physical properties observed for the adsorbent, including the surface area and nitrogen contents. Furthermore, it was found that the lower the dye concentration, the quicker the equilibrium was reached and the lower the capacity of the adsorbent.

As noted in Table 4 and Table 5, in which the kinetic parameters of the four models (PFO, PSO, Elovich, and IPD) are presented, the values of *q_e,_*_exp_ were closer to *q_e_*_,calc_ from PSO than that from PFO. In addition, the coefficient of determination (*R*^2^) indicated the goodness of fit to be in the order of PSO > Elovich > PFO. Comparing the linear and nonlinear models, it was clear that the linear model established a poor *R*^2^ and an overestimation of the capacity compared to the nonlinear equations. This suggested that the nonlinear PSO model was more appropriate for describing the experimental data. Further, the *k*_2_ constant increased with an MO increase, a result that was consistent with the justified thought that the adsorption rate constants are inversely proportional to the initial adsorbate concentration; however, this was not the case for some results, as previously noted [34].

The IPD model is depicted in Figure 8, and the corresponding data are summarized in Table 5. It is clear that the data linearity is poor, indicating less effect of the model on the rate-controlling step. Furthermore, the intercept deviation from the origin suggests another mechanism, such as film diffusion. The overall pattern of the IPD data supports a two-stage mechanism. A first stage with lower values for the intercepts compared to the second stage indicates adsorption controlled by the boundary layers, while the slower rates in the second stage suggest intraparticle diffusion and an equilibrium process. The effect of the IPD mechanism may be higher at a higher *C*_0_ concentration, as revealed by both *C*_1_ and *C*_2_ values being higher for *C*_2_ (Table 5) and increasing as *C*_0_ increased.

#### 3.2.3. Kinetic Studies: Effect of Adsorbent Dose

**Figure 9:**
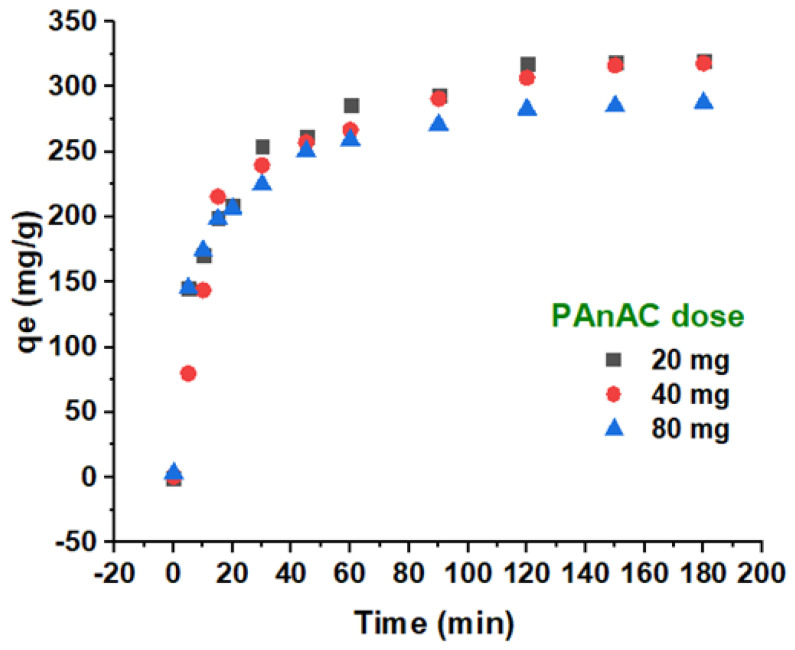
Experimental data plots of adsorption capacity over time at PAnAC dosages of 20, 40, and 80 mg. Conditions: MO adsorbate concentration: 100 ppm; MO volume: 250 mL; adsorbent PAnAC dose: 20, 40, and 80 mg; pH of solutions: 6.4; agitation speed: 150 rpm; temperature: 23 °C; contact time: 0–180 min.

Kinetic studies with variation in the adsorbent dosage (20, 40, and 80 mg per 250 mL of MO solution volume) were also conducted. The adsorption profiles for the kinetic capacity over time are depicted in Figure 9. As can be seen, the adsorption was initially fast in a manner similar to that illustrated in Figure 7, e.g., up to 30 min. Then, adsorption developed slower up to equilibrium. The calculated experimental capacity of PAnAC was found to decrease as the dosage quantity increased, with values of 320.09, 319.73, and 287.73 g/mg when the PAnAC doses were 20, 40, and 80 mg, respectively, as summarized in Table 6.

### 3.3. Adsorption-Based Mechanism

Adsorption kinetics provide insight into the reaction rate and the sorption mechanism, involving mass transfer, diffusion, and the reaction that occurred on the adsorbent surface during adsorption. It is a time-dependent process that consists of several phases, such as (i) an outer diffusion stage during which the sorbate molecules transfer across the liquid film to the adsorbent exterior surface; (ii) intraparticle diffusion, involving the transportation of adsorbate particles from the adsorbent exterior surface to its internal pores; and (iii) the interaction process, with the formation of physical or chemical bonds at the active centers in the pores. However, the interaction step is commonly fast and cannot be treated as rate-limiting [4,35]. Thus, the mechanism is an important descriptor for better understanding the adsorption process. As supposed by data related to kinetic adsorption, the adsorption was mainly PSO. Thus, multilayer coverage and a chemisorption process are supported. The initial sorption rates could be assessed using the PSO and Elovich models through the constants *h* and 𝛼, which were calculated to be 28.5–49.5 and 101.2–223.1 (mg/(g·min)), respectively, and their values increased as MO *C*_0_ increased. The IPD, according to the Weber–Morris model, illustrated that if it is the rate-controlling factor, the plot of *q_t_* vs. *t*^0.5^ will go through the origin. However, the IPD deviation from the origin indicated that IPD is not the sole rate-controlling step and that two processes had taken place [35]. As seen in Figure 8, two linear portions can be identified in the IPD plot, which supports the participation of other mechanisms, including the film diffusion mechanism. Furthermore, IPD intercept *C* showed a faster process in the initial stage of adsorption, as indicated by the smaller *C*_1_ compared to *C*_2_, and both increased as *C*_0_ increased, suggesting a concentration-dependent process. Generally, beside the diffusion process, many other factors can play essential roles in the adsorption process, including the adsorbate structure and the associated functional groups, the textural and surface properties of the adsorbents, and the nature of the adsorbate–adsorbent interaction [36].

**Figure 10:**
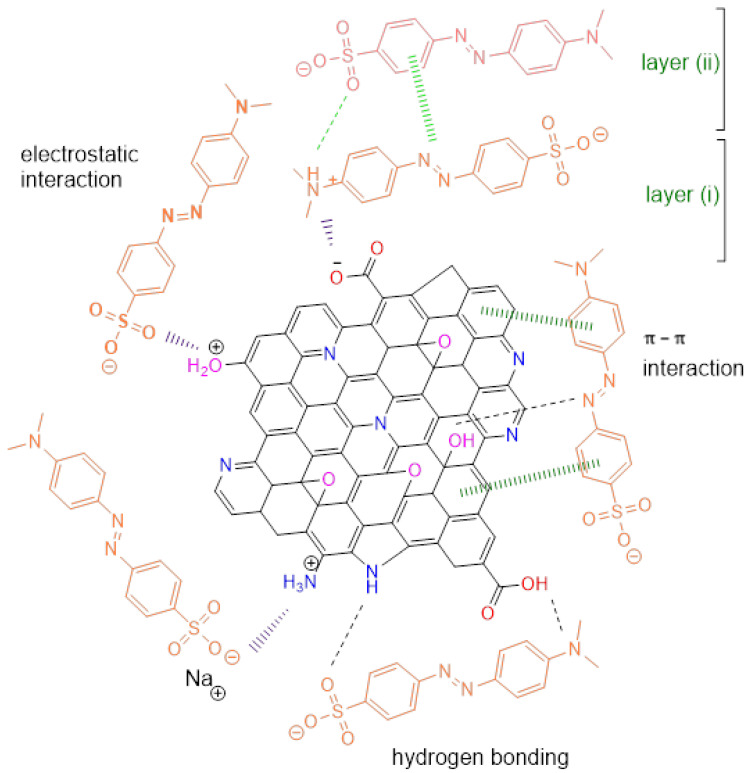
Proposed mechanism for adsorption of MO dye onto PAnAC adsorbent.

The proposed mechanism for MO adsorption onto PAnAC is depicted in Figure 10. As can be seen, the structural properties could support electrostatic attraction the most. The adsorption was performed in the yellow phase of MO at a working solution pH of 7.1. The MO indicator is on its azo-structure, which has a salty structure and is therefore anionic in solution. This is consistent with the results of the pH effect, in which the adsorption decreased as pH increased from 2 to 5. With the solution pH varying from 5 to 7, beside the electrostatics caused by the weak acidic nature of the solution, the molecular form of MO may drive the adsorption capacity increase as well and support the contribution of physical and some other types of adsorption mechanisms (e.g., mechanical) via hydrogen bonding and possible pore tracing events. Above pH 7, the PAnAC surface became more negative, while the OH^−^ molecules became dominant and effectively competed with the anionic MO molecules for adsorption onto PAnAC, leading to a substantial drop in the adsorption capacity at the end.

As in Figure 4, additional peaks emerging between 1150 and 820 cm^−1^ for MO rings confirmed adsorption. The peak at PAnAC 1690 cm^−1^ assigned to C=O stretching (or OH^−^ bending) was down-shifted to 1575 cm^−1^ due to an interaction with MO. This suggests contributions of such functional groups (e.g., C=O, OH, NH, N=N, and N=C) in the adsorption mechanism, and thus chemosorption cannot be ignored. As the adsorption kinetics supposed a PSO mechanism, some MO–MO interactions could be suggested as well. These types of interactions include ionic and π–π interactions, as shown in Figure 10.

Table 7 compares the adsorption performance of PAn and some of the commercial and synthetic activated carbons reported in the literature with PAnAC in terms of their adsorption capacities. For appropriate and easy comparison, adsorbents from the literature were selected based on their similar structures and close adsorption conditions to PAnAC, and their application was also the removal of azo dyes. It is noteworthy that the listed capacities were primarily based on kinetic data. As can be seen, the adsorption performances of commercial ACs in the removal of azo dyes, e.g., reactive violet 5 [37,38] and MO [39], was enhanced after modification, suggesting better performance for synthetic ACs. Furthermore, Khattabi et al. [40] also demonstrated the moderate performance of commercially obtained activated carbon with a capacity (*q_e_*) of 96 mg/g; however, the capacity of PAnAC is seemingly well positioned in the list and surpasses most adsorbents. The adsorption performance of PAn and PAn/metal oxide composites [41,42,43] was low (22–111 mg/g) compared to the listed ACs (commercials: 96–267 mg/g; synthetics: 400–482 mg/g) and PAnAC (406 mg/g). Interestingly, the nitrogen-doped AC revealed a better performance than the undoped one, with capacities of 135 and 120 mg/g, respectively, supporting the argument that activated carbons doped with nitrogen are more active, as nitrogen may provide the AC with additional effective N-based functional groups, thus increasing the number of active sites to adsorb ionic substances, including organic dyes.

## 4. Conclusions

A nitrogen-rich activated carbon (PAnAC) was prepared from a PAn precursor. Its structural and morphological properties, confirmed by FTIR, SEM, BET, TGA, and elemental analysis, evidently support its applicability as an efficient adsorbent for water treatment. The adsorption performance was evaluated in terms of the kinetic behavior of methyl orange (MO) adsorbing onto PAnAC at various MO *C*_0_ values and PAnAC doses. The results indicated better fit to the PSO model than the PFO and Elovich models, suggesting chemisorption and a multilayer process with a rate-limiting step controlled by both film and intraparticle diffusions. Under the applied conditions of pH 7.1, room temperature, and 150 rpm agitation, the kinetic profile revealed fast adsorption over the first 30 min. Then, the rate decreased and reached equilibrium after nearly 60 min. The adsorption capacity (*q_e_*) was found to be solute- and sorbent-dependent, being higher at higher MO concentrations and lower PAnAC dosages. It was optimal at a pH of 7.1, an MO *C*_0_ of 200 ppm, and a PAnAC dose of 40 mg, with a value of 405.6 mg/g. Data of the kinetics favored PSO rather than PFO or Elovich, assuming a chemisorption process on a heterogenous surface, and IPD supported a partial contribution of mass transfer and IPD mechanisms, with a limiting step partially controlled by mass transfer and IPD processes. Therefore, the method of preparation can be extended to similar polymeric wastes for their conversion into valuable materials, including in the water treatment field.

## Figures and Tables

**Table 1:** Linear and nonlinear adsorption kinetic models: PFO, PSO, Elovich, and IPD.

Kinetic Models	Nonlinear		Linear		Constants	Ref.
	Equation	Plot	Equation	Plot		
PFO	$q_{t}=q_{e}\left(1-e^{-k_{1}t\;}\right)$	*qt* vs. *t*	$\ln\left(q_{e}-q_{t}\right)=lnq_{e}-k_{1}t$	*ln*(*q_e_* − *q_t_*) vs. *t*	*k*_1_ (min^−1^)	[24]
PSO	$q_{t}=\frac{k_{2}q_{e}^{2}t}{1+k_{2}q_{e}t}$	*q_t_* vs. *t*	$\frac{t}{q_{t}}=\frac{1}{k_{2}q_{e}t}+\frac{t}{q_{t}}$	*t*/*q_t_* vs. *t*	*k*_2_ (g/mg·min); *h* = *k*_2_*q_e_*^2^ (mg/g·min)	[25]
Elovich	$qt=\frac{1}{\beta }\ln\left(1+\alpha \beta t\right)$	*q_t_* vs. *t*	$qt=\frac{1}{\beta }\ln\left(\alpha \beta \right)+\frac{1}{\beta }lnt$	*q_t_* vs. *lnt*	𝛼 (mg/g·min); 𝛽 (g/mg)	[16]
IPD	-	-	$q_{t}=k_{id}t^{0.5}+C$	*q_t_* vs. *t*^0.5^	*k_id_* (mg/g·min^0.5^); *C* (mg/g)	[26]

**Table 2:** Physicochemical properties of the PAnAC adsorbent.

Material	BET Surface Area (m^2^/g)	Average Pore Width (nm)	CO_2_ Adsorption Capacity (mg/g)	Elemental Analysis (wt%)			
				C	H	N	O *
PAnAC	1893	163.5	56.14	58.80	3.27	18.01	19.92

BET, Brunauer–Emmett–Teller; * calculated values assuming total composition of 100%.

**Table 3:** TGA degradation steps and DTG peaks of Pan, PAnAC, and PAnAC-MO.

Steps	Property	PAn	PAnAC	PAnAC-MO
Step 1	Temperature (°C)	25–153	25–153	25–208
	Mass Loss (%)	7.4	10.9	11.2
	DTG (°C)	95	86	98
	Assig.	Adsorbed volatiles		
Step 2	Temperature (°C)	154–255	154–326	209–474
	Mass Loss (%)	8.2	6.0	14.3
	DTG (°C)	221	248	381
	Assig.	Weak parts and sensitive functional groups		
Step 3	Temperature (°C)	256–419	327–417	-
	Mass Loss (%)	14.7	2.5	-
	DTG (°C)	300	377	-
	Assig.	Oxidative process		-
Step 4	Temperature (°C)	420–686	418–785	-
	Mass Loss (%)	18.9	13.8	-
	DTG (°C)	559	580	-
	Assig.	Carbonaceous step (C. step)		-
Step 5	Temperature (°C)	687–950	-	-
	Mass Loss (%)	7.4	-	-
	DTG (°C)	771	-	-
	Assig.	C. step	-	-
Residue at 950 °C		43.4	64.5	62.3

**Table 4:** Linear and nonlinear plots of PFO, PSO, and Elovich. Conditions: MO adsorbate concentrations: 50, 100, and 200 ppm; MO volume: 250 mL; adsorbent PAnAC dose: 40 mg; pH of solutions: 6.4; agitation speed: 150 rpm; temperature: 23 °C; contact time: 0–180 min.

Conc. (mg/L)	*q_e_*_,exp_. (mg/g)	*PFO*			*PSO*				*Elovich*			Best Fit
		10^−3^*k*_1_ (min^−1^)	*q*_e,calc_. (mg/g)	*R* ^2^	10^−3^ *k*_2_ (min^−1^)	*q_e_*_,calc_. (mg/g)	*h* (mg/(g·min))	*R* ^2^	*𝛼*	*𝛽*	*R* ^2^	
Linear models												
50	264.7	44.6	276.5	0.894	0.43	276.2	32.8	0.998	106.7	0.0216	0.968	PSO
100	319.7	49.5	376.5	0.896	0.35	333.3	38.9	0.997	163.2	0.0190	0.980	PSO
200	405.0	40.7	433.7	0.706	0.29	413.2	49.5	0.997	232.5	0.0158	0.978	PSO
Nonlinear models												
50	264.7	73.6	250.3	0.969	0.37	277.4	28.5	0.995	101.2	0.0214	0.985	PSO
100	319.7	76.1	299.7	0.942	0.33	330.1	36.0	0.984	152.8	0.0178	0.992	Elovich
200	405.0	84.4	368.9	0.957	0.30	405.9	49.4	0.992	223.1	0.0157	0.991	PSO

**Table 5:** The two-stage intraparticle diffusion (IPD) model parameters for the adsorption of MO onto PAnAC adsorbent at MO concentrations of 50, 100, and 200 ppm.

Conc. (mg/L)	*q_e_*_,exp_. (mg/g)	IPD Model					
		Stage 1			Stage 2		
		*K_id1_* (mg/(g·min^0.5^))	*C*_1_	*R* ^2^	*K_id2_* (mg/(g·min^0.5^))	*C*_2_	*R* ^2^
50	264.7	21.16	85.35	0.990	5.91	190.40	0.936
100	319.7	25.74	94.39	0.966	6.69	235.29	0.854
200	405.0	37.46	101.94	0.994	10.49	264.75	0.992

**Table 6:** Experimental data plots of adsorption capacity over time at PAnAC dosages of 20, 40, and 80 mg. Conditions: as given in Figure 9.

Adsorbent	Dose (mg), MO = 100 ppm		
	20 mg	40 mg	80 mg
PAnAC; *q_e,_*_exp_. (mg/g)	320.09	319.73	287.73

**Table 7:** Adsorption capacities of different adsorbents used for removal of various azo dyes.

Adsorbent	Azo Dye Used	Dye Conc. (mg/L)	Capacity (*q_e_*; mg/g)	Mixing Time (min)	Adsorbent Dosage (g/L)	pH	Temp. (°C)	Kinetic Model	Ref.
Commercial activated carbon (CAC; Merck)	Reactive violet 5	1000	246	150	2.5	2	25	Pseudo-second-order	[37]
Acidified cocoa shell activated carbon (ACC-1.0)			400						
Commercial activated carbon (AC; Merck)	Reactive red 120	50	267	180	2.5	2	25	General-order	[38]
*S. platensis* microalgae (SP)			482						
Nitrogen-doped mesoporous carbons (N-OMCs)	Methyl orange	200	135	90	1.0	-	25	-	[39]
Nondoped mesoporous carbons (N-OMCs)			120						
Commercial activated carbon (LOBA Chemie)	Methyl orange	80	96	250	0.75	2	25	Pseudo-second-order	[40]
Polyaniline	Methyl orange	131	63	360	1.2	3	30	-	[41]
Polyaniline nanofibers	Methyl orange	7	22	80	0.5	7	25	Pseudo-second-order	[42]
Polyaniline	Methyl orange	120	111	20	1.0	7	25	Pseudo-second-order	[43]
TiO_2_/Polyaniline composite			119						
PAnAC	Methyl orange	200	406	60	0.16	6.4	23	Pseudo-second-order	This work

## Data Availability

The data that support the findings of this study are included within the article.
